# Human parainfluenza virus 3 fusion protein cleavage: a key determinant of infection and spread

**DOI:** 10.1128/jvi.02126-25

**Published:** 2026-06-03

**Authors:** Kyle Stearns, Tara Marcink, Emily Pawlack, Elizabeth B. Sobolik, Matteo Porotto, Alexander L. Greninger, Stefan Nieweisk, Anne Moscona

**Affiliations:** 1 Department of Pediatrics, Columbia University Vagelos College of Physicians and Surgeons, New York, New York, USA; 2 Center for Host–Pathogen Interaction, Columbia University Vagelos College of Physicians and Surgeons, New York, New York, USA; 3 Department of Physiology & Cellular Biophysics, Columbia University Vagelos College of Physicians and Surgeons, New York, New York, USA; 4 Department of Veterinary Biosciences, College of Veterinary Medicine, The Ohio State University, Columbus, Ohio, USA; 5 Department of Laboratory Medicine and Pathology, University of Washington, Seattle, Washington, USA; 6 Vaccine and Infectious Disease Division, Fred Hutchinson Cancer Research Center, Seattle, Washington, USA; 7 Department of Experimental Medicine, University of Campania “Luigi Vanvitelli”, Caserta, Italy; 8 Department of Microbiology & Immunology, Columbia University Vagelos College of Physicians and Surgeons, New York, New York, USA; Emory University School of Medicine, Atlanta, Georgia, USA

**Keywords:** respirovirus, paramyxovirus, cotton rat, HPIV3, proteases, human parainfluenza virus 3, fusion protein

## Abstract

Human parainfluenza virus 3 (HPIV3) entry into target cells depends on a viral fusion complex composed of hemagglutinin-neuraminidase (HN) and fusion protein (F). In addition to attachment, HN activates F to mediate membrane fusion, while its neuraminidase activity promotes viral release. This creates a biological paradox: virions must repeatedly engage sialic acid-rich substrates in the airway without prematurely triggering F until they reach the target cell sialic acid receptor. Unlike the F proteins of laboratory strains that are cleaved intracellularly by furin to process F from its pro-protein to the active form that is essential for fusion, the F proteins of HPIV3 field strains lack a furin cleavage site and undergo partial cleavage mediated by extracellular serine proteases. We show that virions isolated directly from infected humans contain both cleaved and uncleaved F. In viral migration assays, reducing the proportion of cleaved F on virions increases the distance virions travel before infecting a cell. Even virions bearing only uncleaved F upon egress from infected cells can establish infection *in vivo*. These findings support a model in which uncleaved F on viral surfaces permits virions to engage and disengage sialylated substrates without undergoing premature fusion protein triggering, allowing virions to traverse the airway. Cleavage of F at the target cell then licenses membrane fusion, ensuring that virions initiate infection at the correct site. HPIV3 exploits host protease environments to produce virions with heterogeneity in F cleavage, equipped to either establish local infection or spread to distant target cells.

**IMPORTANCE:**

Parainfluenza viruses employ their surface glycoproteins, the receptor-binding (hemagglutinin-neuraminidase) and fusion (F) proteins, to fuse with target cell membranes and infect the airway. F is made as an inactive precursor that is activated when host cell proteases cleave it to produce the fusion-competent form. Previous studies, utilizing laboratory-adapted viruses, assumed that F proteins are pre-cleaved on virus produced by infected people. We show that virions isolated directly from infected humans contain both cleaved and uncleaved F proteins. In viral migration assays, reducing the proportion of cleaved F on virions increases the distance virions travel before infecting a cell. The uncleaved F proteins enable virions to travel from their cell of origin without undergoing premature fusion protein activation, allowing virions to traverse the airway and infect the proper target cells. HPIV3 exploits host protease environments to produce a spectrum of virions suited to either local infection or intra-host spread.

## INTRODUCTION

Enveloped viruses enter cells by fusing with host membranes. A broad spectrum of human pathogens, such as parainfluenza viruses, coronaviruses, influenza viruses, respiratory syncytial virus, human immunodeficiency virus, and Ebola virus, enter via the Class I fusion mechanism (1–4). These viruses rely on homo-trimeric viral surface fusion glycoproteins that undergo irreversible refolding from a metastable prefusion to a stable post-fusion state, driving membrane merger in the process. For human parainfluenza virus type 3 (HPIV3), a major cause of acute lower respiratory infection, viral entry is mediated by two viral surface glycoproteins: the dimeric multifunctional receptor-binding protein (hemagglutinin-neuraminidase; HN) (5) and the trimeric fusion protein (F) (6–8), which function in a complex on the surface of infectious virions (5, 9). The HN binds to sialic acid-bearing receptors on the target cell surface and triggers F to exit its prefusion conformation and undergo a series of conformational changes that drive membrane fusion (10, 11).

A fundamental unresolved question in paramyxovirus biology is how virions bearing receptor-binding HN proteins can traverse the sialic acid-rich environment of the airway without prematurely triggering fusion proteins. Because receptor engagement by HN activates F, repeated interactions with sialylated substrates in mucus would be expected to activate fusion, and thereby inactivate the fusion mechanism before the virion reaches a target cell (12–15). How virions avoid this premature activation while remaining capable of efficient entry has remained unclear.

Cleavage processing of F from an inactive pro-protein to the active protein is essential for the infectivity of HPIV3 as well as the other paramyxoviruses and pneumoviruses (7). The HPIV3 fusion protein (F) is synthesized as a nonfunctional precursor, F0, which becomes capable of inserting into a target membrane when host cell proteases cleave it into two subunits, F1 and F2, that remain connected by a disulfide bond (16). Cleavage reveals the hydrophobic fusion peptide at the new N-terminus of F1, allowing it to insert into the host membrane as F proceeds to a transient extended intermediate. Subsequent refolding of the fusion protein mediates the merging of viral and cellular membranes, enabling infection of the host cell (2, 17–24). We recently showed that circulating strains of HPIV3 have a conserved monobasic arginine within the F protein cleavage motif that is cleaved by trypsin-like proteases (including TMPRSS2 and TMPRSS13), which are expressed on the surface of a narrow subset of cell types (25). This finding contrasts with the earlier model in which intracellular furin cleaved F0 during trafficking and prior to egress from the infected cell (26–29). This difference suggests that cleavage may not be uniform across virions *in vivo* and raises the possibility that incomplete cleavage plays a functional role. Here, we test the hypothesis that heterogeneity in F cleavage provides a mechanism to resolve the receptor-fusion paradox. We propose that uncleaved F permits receptor engagement without exposure of the fusion peptide, allowing virions to remain infectious during transit, whereas cleavage at an appropriate target cell licenses productive membrane fusion.

The interplay between the properties of HN and F is evolutionarily tuned to maintain virion viability between hosts and to mediate the steps of infection. The receptor-binding, F-triggering, and sialic acid receptor-cleaving functions of HN are in a balance that must govern the outcome of infection (5, 7, 9, 30–35). Mutations in F that affect its inherent “triggerability” and its interaction with HN alter viral fitness in the airway (36–41). We propose that the cleavage state of F on the virus surface provides a regulatory mechanism that determines whether receptor-engaged HN can drive productive fusion. F refolding after exiting the metastable prefusion conformation is irreversible, so viruses have evolved several strategies for preserving F proteins in their prefusion state (5), including, as we propose, the presence of uncleaved F on viral surfaces that can be cleaved at opportune sites for viral entry. Viral fitness may therefore hinge on the precise spatial and temporal control of F cleavage.

To investigate whether F cleavage has a novel regulatory role in infection, we took advantage of our observation that while laboratory strains of HPIV3 can be promiscuously cleaved—including by the ubiquitous furin—field strains strictly conserve the cleavage motif that requires trypsin-like proteases expressed only on the surface of specific cell types. We compared the fusion protein of the lab-adapted virus, which has a dibasic furin cleavage motif containing a lysine at residue 108 (K108), with that of the field strain virus bearing a highly conserved monobasic cleavage site with a glutamate at residue 108 (E108).

Our findings suggest a mechanism for resolving the long-standing question of how virions bearing HN can traverse the sialic acid-rich environment in the airway without triggering the fusion protein until reaching its target. We propose that uncleaved F can be engaged by receptor-bound HN without triggering the exposure of the fusion peptide, allowing virions to remain infectious while navigating the extracellular environment. In this model, virions can be rendered fusion-competent when F is cleaved by a host protease at a target cell. These findings shift the paradigm from one in which HPIV3 fitness depends on uniform fusion complexes tuned to trigger F at a precise time and place to one in which virion populations are inherently heterogeneous. Consequently, variability in F cleavage generates virions with distinct propensities for local infection or distal spread.

## RESULTS

### Human parainfluenza virus 3 collected directly from human subjects carries both cleaved and uncleaved fusion proteins

**Fig 1:**
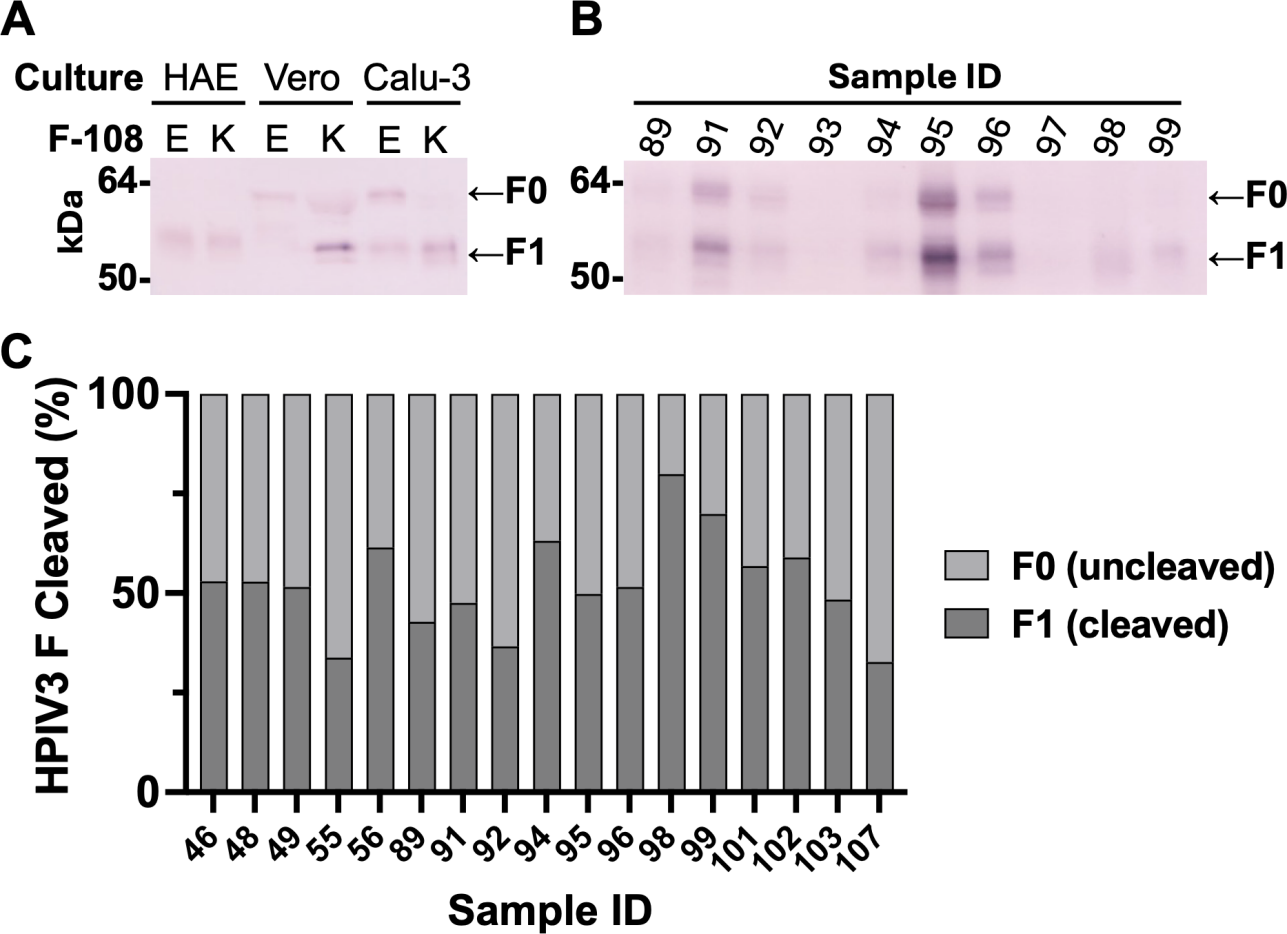
In HPIV3 collected directly from infected human subjects, approximately half of the fusion proteins are cleaved. (**A**) HPIV3 F E108 and HPIV3 F K108 from HAE, Vero, and Calu-3 cells resolved by reducing SDS-PAGE and immunoblotted with an anti-HPIV3 F HRC antibody. (**B**) HPIV3 directly from HPIV3-infected humans without passage was immunoprecipitated with anti-HPIV3 HN antibodies, lysed, resolved by reducing SDS-PAGE, and immunoblotted with an anti-HPIV3 F antibody. (**C**) Densitometry analysis of the HPIV3 F0 (uncleaved) and F1 (cleaved) band intensities.

Field strains of HPIV3 bear the highly conserved monobasic cleavage site F E108, which is not cleaved by furin (25). Field strain F E108 is cleaved variably across different culture models depending on the proteases expressed (25). Human airway epithelial (HAE) cultures and Calu-3 cells that express TMPRSS2 and TMPRSS13 cleave field strain F E108 and release infectious virions. However, most monolayer cell cultures, including Vero cells, lack sufficient trypsin-like protease expression and therefore do not cleave the F E108 from field strains (Fig. 1A) (25, 26). In laboratory-adapted HPIV3 bearing F with the dibasic cleavage site containing K108, most F proteins are cleaved intracellularly by furin (25, 26), allowing the virus to infect cell types promiscuously. During natural infection, HPIV3 infects multiple cell types in the human airway that provide a variety of proteolytic activities (42–45). The dominance of HPIV3 bearing F E108 in circulating field strains of HPIV3 suggests that incomplete F cleavage may confer a fitness advantage.

To determine the extent of HPIV3 F protein cleavage on the surface of viruses from infected people, we evaluated HPIV3 isolated directly from throat swabs and compared the protein cleavage pattern on these virions with that of HPIV3 F E108 and HPIV3 K108 prepared in the laboratory (Fig. 1). Fig. 1A shows the proportions of F0 and cleaved F1 from HAE, Vero, and Calu-3 cells, resolved by reducing SDS-PAGE and immunoblotted with an anti-HPIV3 F antibody. The field strain F E108 is cleaved in HAE and Calu-3, but not in Vero cells. Fig. 1B and Fig. S1 show HPIV3 analyzed directly from HPIV3-infected humans (without passage in cell culture), immunoprecipitated with anti-HPIV3 HN antibodies, lysed, and immunoblotted with an anti-HPIV3 F antibody. Densitometry analysis revealed that 47.6% ± 12.4% of the F proteins are cleaved on the patient-derived virions (Fig. 1C). Samples with no detectable HPIV3 F bands were excluded from this analysis.

### Cleaved HPIV3 F is more readily triggered to extend by receptor-engaged HN than uncleaved F

**Fig 2:**
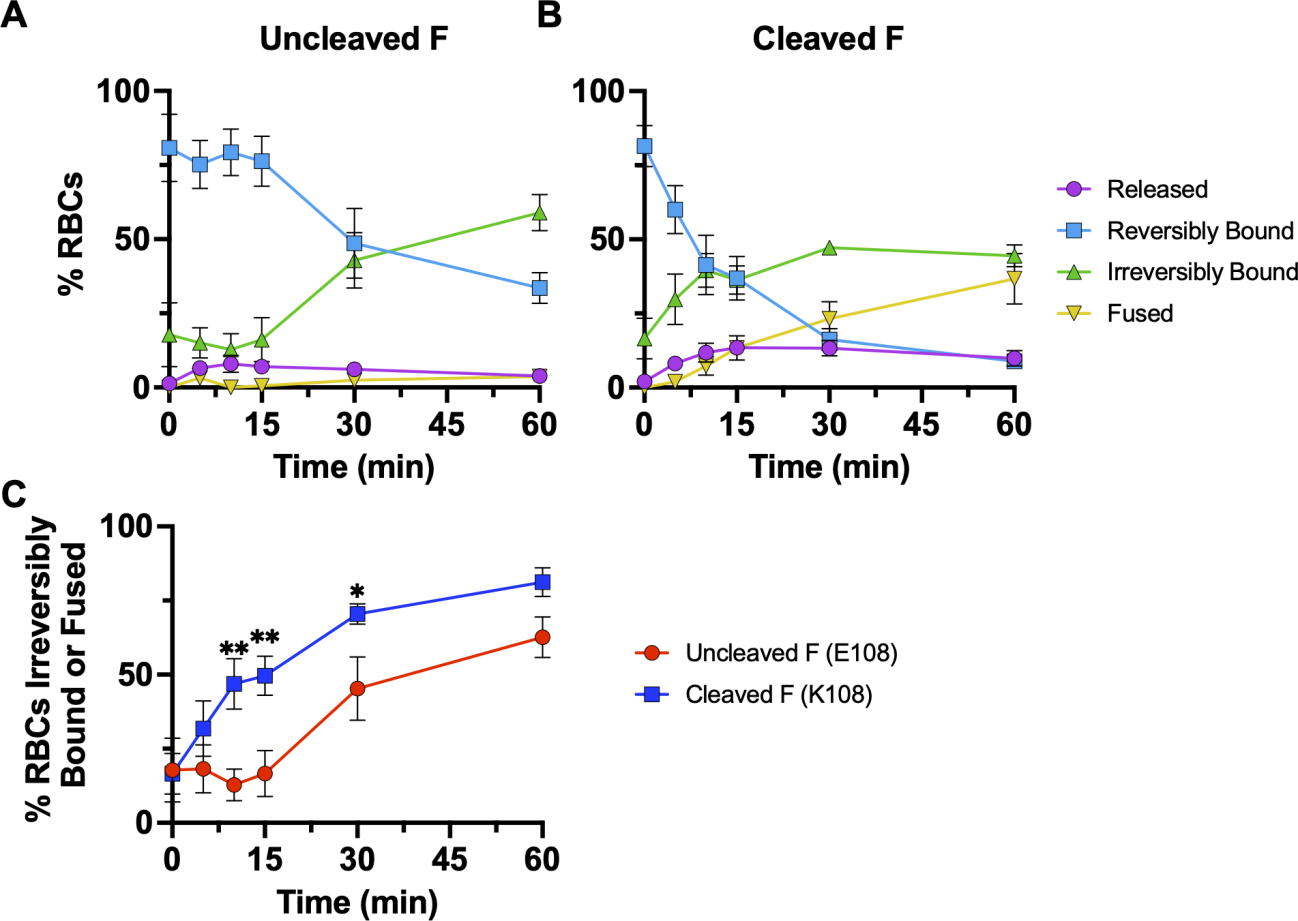
Cleaved F triggers faster than uncleaved F in the presence of receptor-bound HN. Activation of (**A**) uncleaved F or (**B**) cleaved F by receptor-bound HN after 0 to 60 min at 37°C. F triggering measured by the percent of red blood cells (RBCs) released, reversibly bound, irreversibly bound, or fused with the HN-F-expressing cells. (**C**) Summary of percent RBCs fused or irreversibly bound. *P ≤ 0.05, **P ≤ 0.01 by two-way ANOVA and Sidak’s post hoc test. Data are means ± SEM from at least three separate experiments.

Triggering of HPIV3 prefusion F to its extended intermediate state by receptor-bound HN is unidirectional toward refolding to post-fusion F and loss of viral infectivity (10, 14, 15, 30, 46). We hypothesized that, like influenza HA (47), the covalent linkage of uncleaved HPIV3 F1 and F2 restricts uncleaved prefusion F extension relative to cleaved prefusion F. If this is the case, uncleaved prefusion F would be less susceptible to irreversible refolding to its inactive post-fusion conformation. Here, we quantified the steps of HN binding, F triggering, and fusion using an assay that measures the rate at which HEK 293T cells, which lack TMPRSS2 or TMPRSS13 sufficient to cleave F E108 (25), expressing HPIV3 HN and either F E108 (uncleaved) or K108 (cleaved), trigger the extended intermediate of F (11, 48). Cell-surface sialic acid-bearing erythrocytes (red blood cells; RBCs) were bound to cultured monolayer cells co-expressing HN and F at 4°C and then incubated at 37°C for 0 to 60 min to permit F activation. At each time point, we measured the percentage of target RBCs that were either: released into the medium by HN neuraminidase activity (i, purple circles); reversibly bound by HN alone such that Zanamivir (a competitive small molecule sialic acid mimic) releases RBCs (ii, blue squares); irreversibly bound (iii, green triangles), indicating that the triggered uncleaved F complexed with a lipopeptide (VIKI-PEG_4_-Chol) (49) attached to the RBC surface, or that the cleaved F inserted its fusion peptide into the RBC membrane; or fused (iv, yellow triangles) (Fig. 2). Cleaved F is more readily triggered by HN than uncleaved F, as indicated by significantly more RBCs being either irreversibly bound or fused at the 10-, 15-, and 30-minute time points (Fig. 2C).

Triggering of uncleaved F cannot be measured by fusion and was measured using a method we previously established, in which RBC-bound lipopeptides capture the extended triggered fusion proteins and tether the HN-F-expressing cells to the RBCs (46, 49–51). Cells with cleaved F could insert their fusion peptide, be captured by lipopeptide, or fuse, all of which indicate that F was triggered. These results show that cleaved F is more readily triggered than uncleaved F by receptor-engaged HN. As a consequence, delaying F cleavage restricts irreversible triggering of F by sialic acid-bound HN, thereby preserving infectivity during transit.

### The proportion of cleaved HPIV3 F on virions impacts the distance of viral spread from the primary site of infection in cell culture

**Fig 3:**
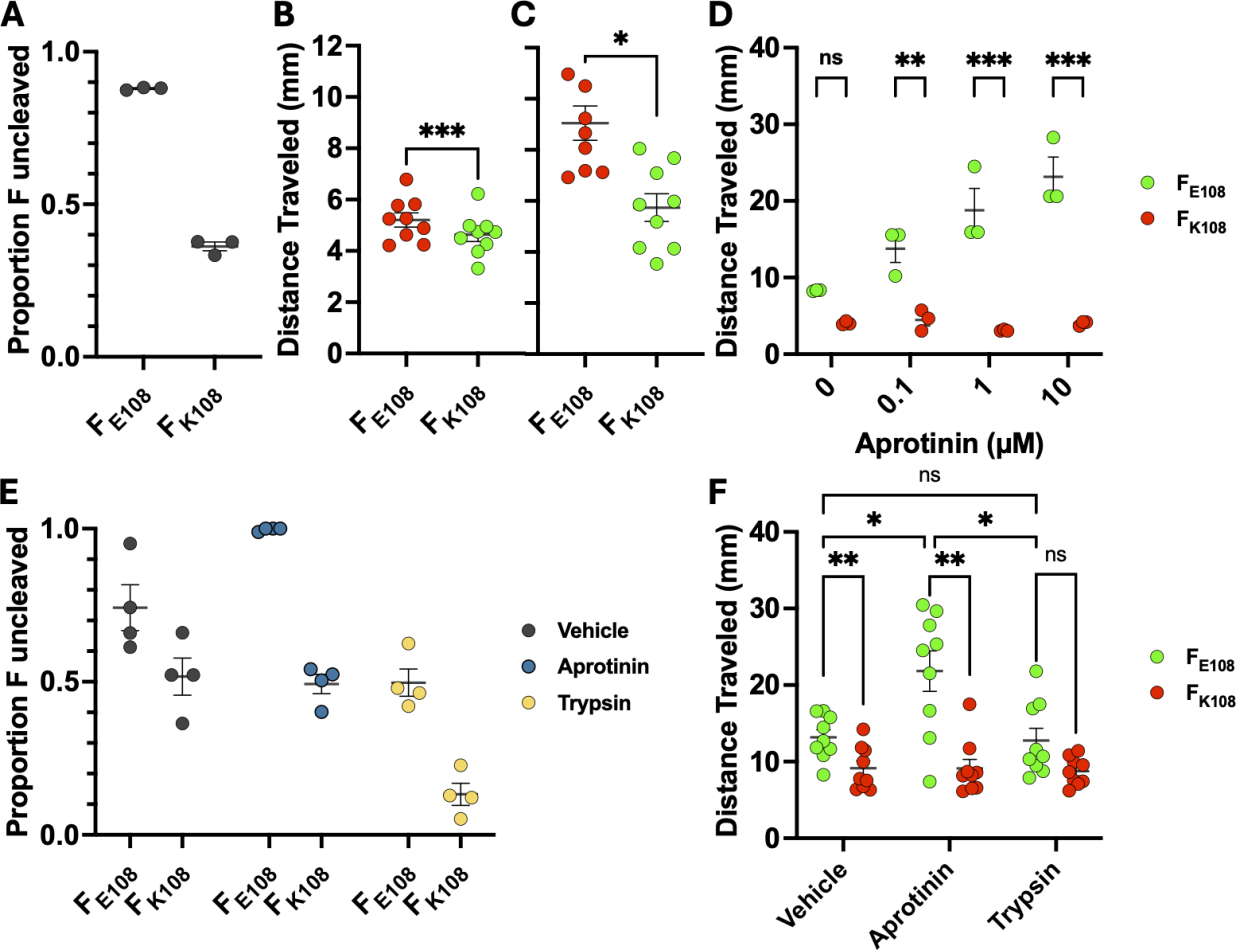
HPIV3 spread distance is inversely proportional to F cleavage. (**A**) HPIV3 F E108 mCherry grown in Calu-3 cells and HPIV3 F K108 eGFP grown in HAE resolved by reducing SDS-PAGE and immunoblotted with anti-HPIV3 F antibody. The proportion of uncleaved F0 was determined by densitometry. (**B, C**) Distance HPIV3 F E108 mCherry and HPIV3 F K108 eGFP traveled from the site of inoculation following (**B**) single entry or (**C**) 5 days of multicycle replication. Proportion of HPIV3 F cleaved. *P ≤ 0.05, ***P ≤ 0.001 by paired *t*-test. (**D**) Distance HPIV3 F E108 eGFP and HPIV3 F K108 mCherry traveled from the site of inoculation following 5 days of multicycle replication in the presence of 0, 0.1, 1, or 10 µM aprotinin. **P ≤ 0.01, ***P ≤ 0.001 by two-way ANOVA and Holm-Sidak’s post hoc test. (**E**) HPIV3 F E108 eGFP and HPIV3 F K108 mCherry grown in Calu-3 in the presence of vehicle, 10 µM aprotinin, or 0.1 µg/mL TPCK-treated trypsin were resolved by reducing SDS-PAGE and immunoblotted with anti-HPIV3 F antibody. The proportion of uncleaved F0 present in virus solutions was determined with densitometry. (**F**) Distance HPIV3 F E108 eGFP and HPIV3 F K108 mCherry traveled from the site of inoculation following 5 days of multicycle replication in the presence of vehicle, 10 µM aprotinin, or 0.1 µg/mL TPCK-treated trypsin. *P ≤ 0.05, **P ≤ 0.01 by two-way ANOVA and Holm-Sidak’s post hoc test. Values are represented as means and ± SEM from at least three biological replicates.

Based on the finding in Fig. 1 that about half of the fusion proteins on circulating HPIV3 are cleaved, we hypothesized that F protein cleavage may affect the fraction of fusion-ready HN-F complexes on viral surfaces and thereby modulate viral spread. To assess the effect of the proportion of cleaved F on virion spread, we designed a novel experimental procedure to monitor the distance that virions travel before infecting cells. To achieve this, we utilized transparent ibidi µ-Slide I Luer slides, which have 0.4 mm thick tissue culture-treated channels flanked by two ports. This culture setup enables the addition of virions to one of the ports and the subsequent measurement of virion spread from the port, as indicated by the appearance of the respective fluorescent markers of the recombinant HPIV3 F E108 mCherry or HPIV3 F K108 eGFP. The proportion of F cleaved on the input virions was quantified by western blot analysis (Fig. 3A). The HPIV3 F E108 stock virus solutions had about 10% cleaved F, whereas the HPIV3 F K108 stock virus had >60% cleaved F (Fig. 3A).

Viral spread distance from the point of inoculation to the site of infected cells was first measured after a single round of infection, reflecting entry only. An equal number of infectious HPIV3 F E108 mCherry and HPIV3 F K108 eGFP virions were added to channel slides seeded with Vero cells for 90 min at 37°C, and then the slides were flushed with Zanamivir to detach viruses bound by HN alone (52) and prevent further entry. HPIV3 F E108 traveled 12% farther than HPIV3 F K108 (Fig. 3B), with a mean difference of 0.57 mm (Fig. 3B). Viral travel during the course of multicycle replication was measured by infecting channel slides seeded with Calu-3 cells—a cell line that supports the production of infectious HPIV3 F E108—and allowing viruses to spread for 5 days after infection. Under these conditions, HPIV3 F E108 spread 57% farther on average relative to HPIV3 F K108 (Fig. 3C; *P* < 0.01).

To determine whether HPIV3 spread is modulated by the proportion of cleaved F on virions, we used the serine protease inhibitor aprotinin, which we have shown to inhibit HPIV3 F E108 cleavage in a dose-dependent manner while having no effect on HPIV3 F K108 (Fig. 3D) (25). Channel slides seeded with Calu-3 cells were coinfected with equal titers of HPIV3 F E108 eGFP and HPIV3 F K108 mCherry and allowed to spread for 5 days in the presence of 0, 0.1, 1, and 10 µM aprotinin. HPIV3 F E108 spread significantly more than HPIV3 F K108 with 0.1 (*P* < 0.01), 1 (*P* < 0.001), and 10 µM (*P* < 0.001) aprotinin treatment (Fig. 3D). No significant difference (*P* = 0.08) was observed without aprotinin treatment in this experiment, likely due to the relatively small sample size compared to the experiment in Fig. 3C. The HPIV3 F K108 virions traveled a similar distance irrespective of the amount of aprotinin present, suggesting that inhibiting extracellular cleavage of F promotes HPIV3 F E108 spread. To assess whether, conversely, cleaving the F on HPIV3 F E108 virions would decrease travel distances, HPIV3 F E108 eGFP and HPIV3 F mCherry were added to the same port and then allowed to spread for 5 days in the presence of vehicle, 10 µM aprotinin, or 0.1 µg/mL TPCK-treated trypsin to cleave F. Trypsin treatment increased the proportion of F cleaved on the HPIV3 F E108 virions (Fig. 3E) and reduced their mean travel distance to that of HPIV3 F K108. We did not detect a statistically significant reduction in HPIV3 F E108 travel distance in the presence of trypsin relative to the vehicle control, but we would theorize that a similar study with greater statistical power would observe reduced viral travel distance because of increased F cleavage by trypsin. In the presence of vehicle or aprotinin, HPIV3 F E108 traveled significantly (*P* < 0.01) farther than HPIV3 F K108 (Fig. 3F). Taken together, these data indicate that the proportion of cleaved F determines whether virions are biased toward immediate entry or continued transit prior to infection. Virions bearing predominantly uncleaved F are less prone to productive triggering during receptor engagement and can facilitate distal spread, whereas virions bearing more cleaved F are primed for fusion upon encountering neighboring target cells.

### Uncleaved HPIV3 F0 can be triggered by receptor-engaged HN to mediate cell-cell fusion

**Fig 4:**
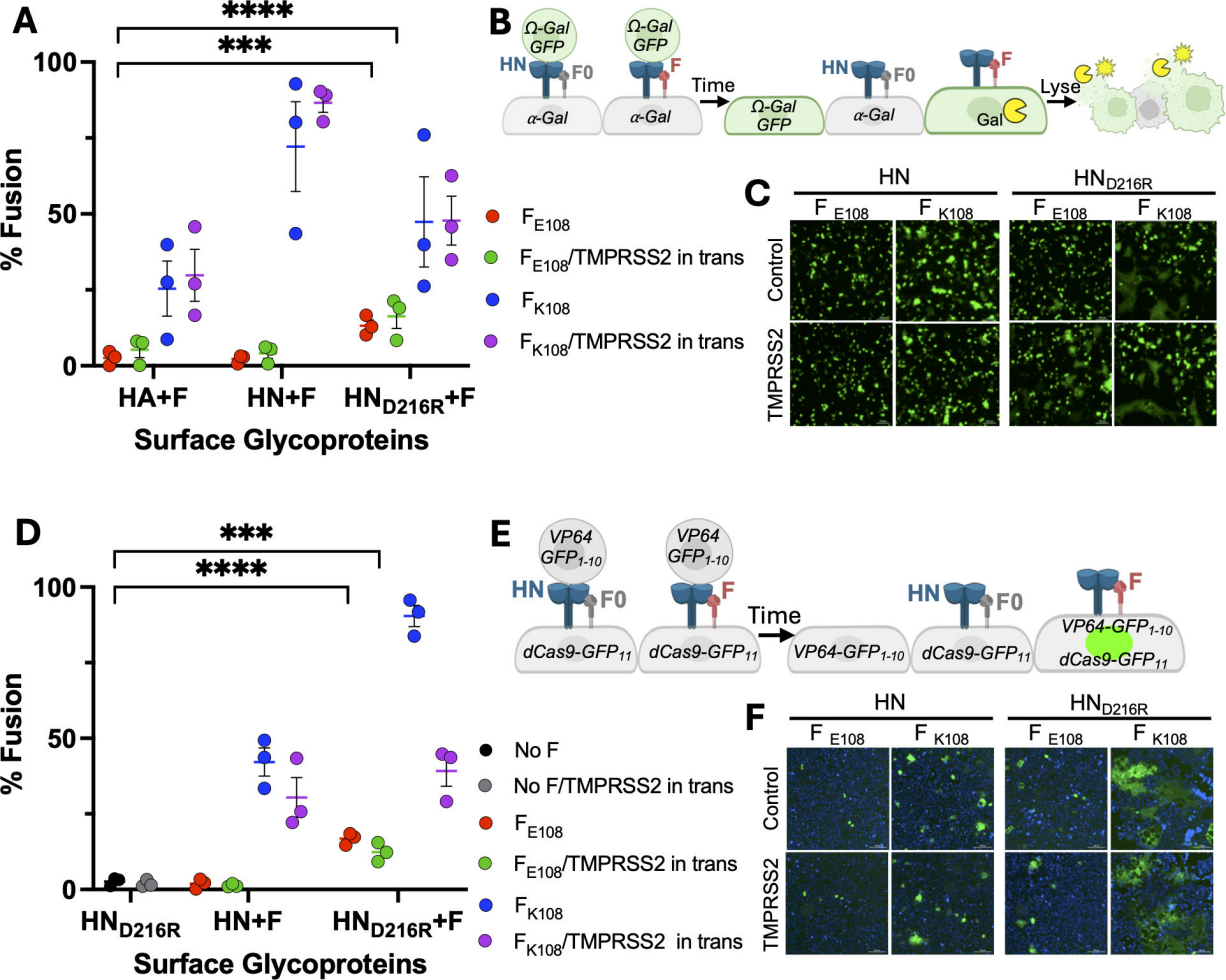
HPIV3 F E108 mediates cell-cell fusion when paired with an HN that continually engages receptor. Percent fusion mediated by influenza HA, HPIV3 HN, HPIV3 HN D216R expressed with no F (black), no F targeting cells expressing TMPRSS2 *in trans* (gray), F E108 (red), F E108 targeting cells expressing TMPRSS2 *in trans* (green), F K108 (blue), F K108 targeting cells expressing TMPRSS2 *in trans* (purple). (**A**) Fusion activity measured with β-galactosidase complementation assay. (**D**) Fusion activity measured with VP64-GFP_1-10_ and dCas9-GFP_11_ complementation assay. (**B, E**) Schematic of complementation assays. (**C, F**) Representative images 16 h after overlaying target cells on viral surrogate cells. ***P ≤ 0.001, ****P ≤ 0.0001 by one-way ANOVA and Dunnett’s post hoc test. Data are means ± SEM from at least three separate experiments.

The presence of uncleaved F on HPIV3 virions (Fig. 1) suggests that viruses may extend infectivity by carrying both fusion-ready cleaved F and premature triggering-resistant uncleaved F. We asked how virions bearing uncleaved F are induced to enter cells upon arrival to a suitable target. We had previously observed that TMPRSS2 or TMPRSS13 expression on target cells (*in trans*) was not sufficient to promote fusion between HN-F E108-expressing cells and receptor-bearing cells (25). Here, we hypothesized that upon HN’s receptor engagement, the cleavage site of the corresponding F may be exposed and available to proteases. To explore this possibility, we paired F with a previously characterized neuraminidase-dead HN (HN D216R (53) that does not detach from target cell receptors and therefore provides continual activation of F (Fig. 4). With this HN/F pair, we used several distinct fusion assays to determine if HPIV3 F E108 can mediate fusion upon interaction with a target cell, without exogenous protease. The β-galactosidase complementation system used here is the same that we used previously to dissect viral fusion mechanisms (48, 53). In a second assay, cells expressing HN, F, and a subunit of the complement system were overlaid with HEK293/dCas9-VP64 + MPH target cells expressing scrambled or TMPRSS2 gRNAs and the complementary subunit of the complement system for detection. Finally, we developed fluorescence-based complementation assays here to enable direct visualization of cell-cell fusion. In all fusion assays, the influenza hemagglutinin (HA) is a control receptor-binding protein that binds sialic acid receptor but does not activate HPIV3 F (10, 11, 31).

The cell-cell fusion assay (Fig. 4A) is quantitated when overlaid GFP-expressing target cells form green fluorescent syncytia or when catalytically active β-galactosidase is generated when fusion unites the alpha and omega β-galactosidase subunits. Multicellular syncytia were visible under all conditions with either F E108 or F K108 expressed with the neuraminidase-dead HN (HN D216R) (Fig. 4C). Fusion was increased when HN D216R was paired with F E108, with or without TMPRSS2 expressed on the target cell, relative to F E108-mediated fusion in the presence of control HA (Fig. 4A). The observation that HN-F K108 pair fusion values are higher than those of the HN D216R-F K108 pair, despite larger syncytia with HN D216R (Fig. 4A), may be explained by syncytium formation decreasing cell viability and expression of the β-galactosidase subunits.

The fluorescence-based complementation assays enable direct visualization of cell-cell fusion through the complementation of individually nonfluorescent subunits of VP64-GFP_1-10_ with dCas9-GFP_11_ to form green fluorescence in the nuclei of fused cells (54) (Fig. 4E). For F K108, intense fusion was observed when paired with either standard HN or HN D216R (Fig. 4F). Fig. 4D shows evidence that HN D216R paired with F E108, with or without TMPRSS2 expressed on the target cell, permits fusion. In this assay, HN D216R alone and 621 nM 4C06 VHH-Fc (25, 55) (a neutralizing anti-HPIV3 prefusion F antibody) were included to confirm that receptor engagement was not sufficient for cell-cell fusion and that fusion was blocked if F was prevented from refolding by 4C06 VHH-Fc (Fig. S4).

These data suggest that uncleaved F in the HN-F complex can proceed to mediate cell-cell fusion without an exogenous protease. The HA + F E108 control illustrated that F E108 requires triggering by HN—not simple receptor tethering—to mediate fusion. Expression of TMPRSS2 *in trans* appeared to induce larger syncytium formation, but these syncytia appeared to reduce signal from the complementation systems likely due to decreased viability (Fig. 4D). The HN D216R-only control confirmed that continuous HN receptor engagement alone is not sufficient to mediate fusion in the absence of F. Inhibition of F E108 + HN D216R by 4C06 VHH-Fc confirmed the requirement for functional F for fusion. Taken together, these data show that continual triggering of F E108 by receptor-engaged HN D216R allows fusion to occur, suggesting that the triggered conformation of uncleaved F E108 exposes the cleavage motif to HEK cell endogenous proteases. In this model system, this cleavage event is rare, with little F E108-mediated fusion relative to that of F K108.

### F0-bearing HPIV3 infects *in vivo*

**Fig 5:**
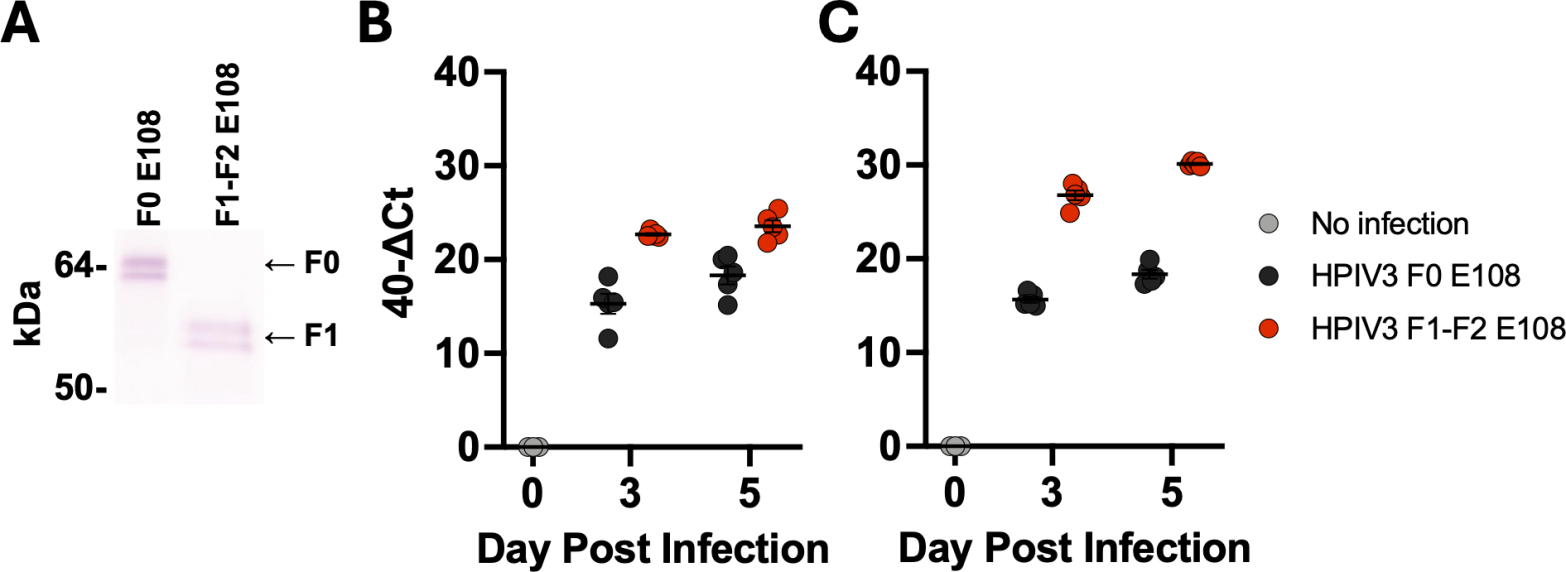
HPIV3 bearing only F0 can infect cotton rats. Cotton rats were intranasally inoculated with 1.5 × 10^5^ PFU of HPIV3 F E108 alone (F0 E108) or HPIV3 F E108 treated with TPCK-treated trypsin (F1-F2 E108). Five animals were included in each infected group, and three were included in the uninfected group. Nasal and lung tissue homogenates were collected 3 or 5 days post-infection. (**A**) HPIV3 F E108 and HPIV3 F1-F2 E108 from Vero cells resolved by reducing SDS-PAGE and immunoblotted with an anti-HPIV3 F antibody. (**B and C**) HPIV3 RNA levels in nasal (**B**) or lung (**C**) homogenates detected with RT-qPCR. A significant increase in HPIV3 gene expression was detected in all HPIV3 inoculated animals relative to no infection controls with *P* ≤ 0.0001 determined by one-way ANOVA and *P* ≤ 0.0001 determined by Dunnett’s post hoc test for each infection group compared to the control no infection group. Data are means ± SEM from at least three animals per group.

The results shown in Fig. 4 suggest that there is a mechanism for cleavage of HPIV3 F E108 that permits entry by virions bearing uncleaved fusion proteins. To determine whether HPIV3 bearing uncleaved F (HPIV3 F0 E108) mediates infection *in vivo*, cotton rats were intranasally inoculated with HPIV3 derived from Vero cells (which do not cleave HPIV3 F E108; Fig. 5A). Cotton rats were infected in parallel with the same virus stocks treated with trypsin to cleave their surface F proteins. HPIV3 RNA was detected in both nasal (Fig. 5B) and lung (Fig. 5C) homogenates at 3 and 5 days post-infection with HPIV3 F E108 alone (F0 E108) or HPIV3 F E108 treated with trypsin (F1-F2 E108), indicating that HPIV3 F0 E108 established infection in cotton rat upper airways and lungs (Fig. 5B and C).

## DISCUSSION

HPIV3 F cleavage is a key regulator of HPIV3 fusion activity and contributes to the multitude of strategies employed by the virus to ensure that activation of the entry mechanism occurs only at the right time and place (5, 15, 25, 34, 36–41, 56). The HN and F may be thought of as a catalyst and reactant, respectively, and HPIV3 genome survival is contingent on the interplay of these proteins permitting cell entry before infectivity is lost due to depletion of all F proteins by premature triggering. The multifunctional HN catalyzes binding and cleavage of sialic acid receptors, maintains F in its prefusion state, and upon sialic acid receptor engagement transmits a triggering signal to F (5, 10, 31, 36, 37, 39–41). As the reactant, F proteins must remain in their prefusion conformation to be fusion-competent, since activation results in irreversible refolding to an inactive post-fusion state. The finding that field strains of HPIV3 do not replicate in most monolayer culture cells led us to discover that F protein processing is highly selective for a limited set of cells (25, 36) and suggested that regulation of F protein cleavage may modulate viral spread.

The dominance of the serine protease-limited HPIV3 F cleavage site in circulating strains (25, 57) suggested that a fitness advantage is conferred by regulating F cleavage. In contrast to efficiently cleaved laboratory-adapted F proteins, only half of the F proteins were cleaved on virions from human donors, showing that HPIV3 in circulation bears both fusion-ready cleaved F proteins and uncleaved F. We considered the possibility that uncleaved HPIV3 F may prevent fusion protein depletion by maintaining uncleaved fusion proteins. In the case of influenza virus, the uncleaved precursor of the fusion protein (HA_0_) was triggered by low pH without proceeding to membrane insertion and reverted to its prefusion conformation upon return to neutral pH (47). We assessed whether uncleaved HPIV3 F0 may be more resistant than cleaved F protein to irreversibly exiting its prefusion conformation, either through covalent tethering of the fusion peptide to a target membrane (46) or through return to its prefusion conformation after removal of the triggering stimulus (heat exposure). Surprisingly, the cleavage state of F on virions did not alter the environmental degradation rate of virions in the absence of receptor engagement (Fig. S2) or the inherent stability of the F protein alone (Fig. S3). However, when in complex with receptor-bound HN, uncleaved F took twice as long to extend and irreversibly bind to target cell membranes, relative to otherwise identical fusion machinery with cleaved F. This observation suggests that uncleaved F is less likely to undergo triggering upon HN receptor engagement and therefore may be less vulnerable to virucidal premature activation by the receptor mimics, such as mucins, in the human airway (12, 13, 15).

The utility of uncleaved F proteins for viral fitness is based on a mechanism of F cleavage by proteases on the surface of or proximal to target cells. Although we have previously reported that TMPRSS2 and TMPRSS13 cannot cleave HPIV3 F E108 in *trans* (25), we show here that F E108 can mediate cell-cell fusion when continually triggered by a neuraminidase-dead HN. We speculate that the extension of F, upon HN’s stable receptor engagement, may render the fusion peptide loop susceptible to protease cleavage. This rare event may provide an alternative entry mechanism through which uncleaved prefusion F proteins maintain viability until receptor engagement and preferentially infect cells with sufficient proteases to cleave triggered F. HPIV3 entry via F cleavage occurring only in the target organism was demonstrated by the infection of 10 of 10 cotton rats inoculated with HPIV3 containing uncleaved F E108. Reduced HPIV3 gene expression was observed in the group infected with HPIV3 containing uncleaved F E108 relative to cotton rats infected with HPIV3 containing cleaved F. This result aligns with previous studies showing that Sendai virus, which also has an F that is cleaved by trypsin-like proteases after a monobasic arginine, infected mice inoculated with virions bearing only F0 (58, 59). Taken together, these observations suggest uncleaved F may be resistant to premature activation and can mediate entry into a naïve host, thereby initiating exponential spread of HPIV3.

If virion spread away from their point of origin explains why serine protease-cleaved HPIV3 F proteins (F E108) dominate over furin-activated proteins (F K108) in the field, this dominance may result from altered F cleavage. Our results indicate that viral displacement from the site of infection was inversely proportional to the magnitude of fusion protein cleavage during either single entry or multicycle replication. We speculate that virions with less-cleaved F have a lower probability of insertion per HN engagement with receptor and thus can traverse more cells and more of the airway before being sequestered by infection. In distinction to HPIV F, the influenza viral fusion protein HA is activated intracellularly by acidic pH. Since the HPIV3 HN pairs receptor binding with fusion activation, this feature—if unregulated—would deplete the finite F proteins through binding to target cell mimics (e.g. mucins) and lead to viral sequestration through infection of neighboring cells, thereby limiting viral spread.

Our findings provide a potential resolution to this longstanding paradox in paramyxovirus biology. Uncleaved Fs on HPIV3 virus surfaces are resistant to premature triggering when HN engages target cell mimics, promote travel away from the origin of infection, and can subsequently mediate entry following cleavage at the target cell surface. The absence of a furin cleavage site and resistance to processing by this ubiquitous protease leads to a spectrum of virions variably equipped to either establish local infection or to maintain viability during transit and spread to more distant target cells.

## MATERIALS AND METHODS

### Cells and viruses

Vero (ATCC) and HEK 293T (ATCC) cells were grown in Dulbecco’s modified Eagle’s medium (DMEM) (Gibco) supplemented with 10% fetal bovine serum (FBS) and 1% penicillin-streptomycin (P/S) (Gibco) at 37°C in 5% CO2. Calu-3 (ATCC) cells were grown in Eagle’s minimum essential medium (ATCC) supplemented with 10% FBS and 1% P/S (Gibco) at 37°C in 5% CO2.

For infection and production of viruses, EpiAirway AIR-100 system (MatTek Corporation), Vero (ATCC), or Calu-3 (ATCC) cells were infected with an MOI of 0.01–0.1 of HPIV3 for 90 min. Virions released by the cells were titered by limiting dilution infection of Vero cells.

### Recombinant virus growth and purification

Recombinant viruses were generated by reverse genetics as previously described (40, 44) using an HPIV3 clinical isolate (CI-1) background harboring single mutations in F (E108 or E108K) and a recombinant eGFP or mCherry cassette between genes P and M. Resulting viruses were propagated using the HAE EpiAirway AIR-100 system (MatTek Corporation) and Calu-3 (ATCC). Viruses were titered by limiting-dilution infection of Vero cells (ATCC), and infected cells were quantified using a Cytation5 (Agilent). All recombinant viruses were sequenced using metagenomic next-generation sequencing (60). All experiments comparing HPIV3 F E108 and HPIV3 F K108 were performed using viruses with identical genomes differing only at residue 108 and in the fluorescent marker cassette.

### HPIV3 genome sequencing

Shotgun RNA sequencing metagenomic reads (60) were adapter- and Q20 quality-trimmed using Trimmomatic v.0.39 (61). Reads for the viral genome were aligned to the reference sequence for the HPIV3 expressing mCherry (GenBank accession no. OP821798) using bwa-mem v.0.7.17-r1188 (https://arxiv.org/abs/1303.3997), and variant allele frequencies were extracted using bcftools v.1.9 (84) and annotated using VarScan v.2.3 (62) (BioProject PRJNA1132297).

### HPIV3 enrichment and immunoblotting

De-identified HPIV3-positive clinical isolates from throat swab material were obtained from the clinical microbiology laboratory of Dr. Stephen Jenkins at Weill Cornell Medical Center. HPIV3 in 100 µL of each clinical sample was immunoprecipitated with Dynabeads Protein G (Invitrogen) bound to rat anti-HPIV3 HN (10F7, Genovac), and the viral proteins were solubilized with RIPA lysis buffer (Fisher Scientific). The proportion of HPIV3 F cleaved on virions was determined by resolving the viral proteins on 4%–20% Tris-glycine SDS-PAGE (Novex) under reducing conditions, transferring the proteins with an iBlot2 NC stack (Invitrogen), and then detecting F proteins with rabbit anti-HPIV3 F HRC antibody (Genscript) and WesternBreeze Chemiluminescent Kit (Invitrogen). For quantitative analysis, raw immunoblot images were imported into Fiji (ImageJ version 2.9.0/1.53t). Lanes of interest and density profiles were analyzed using the Gels analysis tool. The area under the peak for each band was calculated with baseline subtraction to account for background signal.

### β-Galactosidase complementation fusion assay

Cell-cell fusion was quantified by measuring β-Gal complementation as performed previously (48, 53). Receptor-bearing HEK 293T (ATCC) cells expressing the omega peptide of β-Gal, GFP, and control or TMPRSS2 CRISPRa plasmid (Santa Cruz Biotechnology) were mixed with HEK 293T cells co-expressing envelope glycoproteins (HN and F [E108 or K108]) and the α peptide of β-Gal. Cell-cell fusion was quantified by measuring galactosidase activity in fused cell lysates (Galacton-Star substrate; Applied Biosystems, T1012), and luminescence was read after 1 hour on a Tecan M1000 Pro.

### Virus environmental stability assay

The viability of virions incubated *ex vivo* was determined by incubating HPIV3 F0 E108 mCherry or HPIV3 F K108 mCherry for 24 h at 4, 22, or 37°C either in solution (PBS) or in a dried state. Virions for the “dry” incubations at 22 and 37°C were dried in sealed containers with anhydrous CaCl_2_ for 3 and 1 hours, respectively, at their incubation temperatures. Virions for “dry” incubation at 4°C were lyophilized for 15 min. Following 24-hour incubation, dried virions were rehydrated with deionized water to the original volume. Virion solutions were titered by limiting dilution infection of Vero cells in the presence of 0.1 µg/mL TPCK-treated trypsin during a 90-minute infection.

### F stability assay

HEK 293T cells were transiently transfected with HPIV3 F E108 or HPIV3 F K108 for 18 h at 37°C. Cells were either kept at 4°C or transferred to 55°C for 15, 30, or 60 min. After incubation, the cells were washed, and 1 µg/mL of anti-HPIV3 prefusion F conformation-specific antibodies, PIA174 (63) or 3 × 1 (64) mAb, were added to all wells for 60 min on ice to detect loss of the prefusion state. After washing, cells were stained with anti-human antibody conjugated with DyLight 594 (0.5 µg/mL) (Abcam) for 60 min at 4°C. Cells were imaged on a Cytation 5 (Agilent), and fluorescent intensity was calculated. The data were normalized to the fluorescence at 4°C for each condition.

### Measurement of F-activation and fusion between RBCs and envelope glycoprotein-expressing cells

HEK 293T cells were transiently transfected with HPIV3 HN D216R and HPIV3 F bearing E108 or K108 for 18 h at 37°C. Cells were treated overnight with 25 mU/well of exogenous neuraminidase (Sigma Aldrich) to deplete sialic acid receptors and then washed and incubated with 1% RBC suspensions (pH 7.5) for 30 min at 4°C. After the samples were washed to remove unbound RBCs, they were treated with 4 µM VIKI-PEG_4_-Chol, a cholesterol-conjugated HRC-derived peptide that inserts into the cell membrane and binds to the extended immediate of HPIV3 F (46), in pH 6 CO_2_-independent medium (Gibco) and brought to 37°C for 0, 5, 15, 30, or 60 min. The plates were rocked, and the liquid phase collected in V-bottom plates for measurement of released RBCs. The cells were incubated with 10 mM zanamivir in CO_2_-independent medium to release RBCs that were attached via HN receptor engagement. The liquid phase collected in V-bottom plates for measurement of reversibly bound RBCs. Plates were spun down, and pelleted RBCs were lysed in milli-Q water and transferred to a flat-bottom 96-well plate for quantitation. The cells were then incubated with RBC lysis solution (ammonium-chloride-potassium lysis buffer; Thermo Fisher Scientific), where the lysis of unfused RBCs removed cells that were attached only via F (bound to inhibitory lipopeptide). The liquid phase collected in flat-bottom plates for measurement of irreversibly bound RBCs. The cells were then lysed in 200 μL of dodecyl maltoside HEPES (DH) buffer (5 mM Hepes, 10 mM NaCl, and dodecyl maltoside [0.5 mg/mL]) 1:10 in PBS and transferred to flat-bottom 96-well plates for quantification of fused RBCs. Hemoglobin absorbance for the above four compartments was determined by measuring absorbance at 405 nm on a Tecan M1000 Pro. The percentage of irreversibly bound and fused RBCs was calculated by dividing the sum of irreversibly bound and fused absorbance by the sum of released, reversibly bound, irreversibly bound, and fused RBCs.

### Fluorescent complementation fusion assays

Cell-cell fusion was quantified by measuring GFP complementation. Receptor-bearing HEK293/dCas9-VP64 + MPH cells were transfected with scrambled gRNA or TMPRSS2 CRISPRa gRNA and dCas9-GFP_11_. Viral surrogate HEK293T cells were transfected with HPIV3 envelope glycoproteins (HN or HN D216R, and F E108 or F K108) and VP64-GFP_1-10_ and incubated in the presence of 10 mU neuraminidase per well in a 96-well plate. Following 24-hour incubation, viral surrogate cells were washed to remove residual neuraminidase, and target cells were overlaid onto viral surrogate cells. Cells were incubated overnight at 37°C to permit fusion. Cells were imaged on a Cytation 5 (Agilent), and fluorescent intensity was calculated. Cell-cell fusion was quantified by measuring the total fluorescent area. Percent fusion was quantified by comparing relative fluorescence of each condition to HN D216R-F K108.

### Viral channel migration assay

Viral migration from the site of inoculation was measured by quantifying the distance of fluorescent infected cells from the well port of infection. μ-Slide I^0.4^ Luer slides were seeded with Vero cells (single entry) or Calu-3 cells (multicycle replication). Slides were co-infected with equal titers HPIV3 F E108 and HPIV3 F K108 for 90 min at 37°C. Following infection, the inoculation solutions were flushed out of the channels via addition of media to the port opposite the site of infection and aspiration of media from the port of infection. For single-entry experiments, the channels were flushed with media containing 5 mM Zanamivir after the inoculation period to halt subsequent infection. Slides were imaged 24 h post-infection. For multicycle replication experiments, the inoculation solution was flushed three times with Opti-MEM and replaced with Opti-MEM containing no treatment, 0.1–10 µM aprotinin, or 0.1 µg/mL TPCK-treated trypsin where listed. At 5 days post-infection, slides were imaged to quantify the distance virions traveled from the port of infection. Entire channels were imaged on a Cytation 5 (Agilent), and Gen5 software was used to quantify fluorescent cell distances from the inoculation port.

### Cotton rat infections

Inbred cotton rats (*Sigmodon hispidus*) were purchased from Inotiv (formerly Envigo; West Lafayette, IN). All cotton rats were maintained in NexGen Rat 900 polysulfone microisolator cages (Allentown Inc., Allentown, NJ) in a barrier facility with 12:12 h light cycles, 22 ± 2°C, and 50–70% relative humidity and were used at 8–10 weeks of age.

Cotton rats were intranasally inoculated with 1.5 × 10^5^ PFU of HPIV3 F E108 without (F0 E108) or with TPCK trypsin treatment (F1-F2 E108) under isoflurane narcosis. Three or 5 days post-infection, animals were euthanized by C0_2_ inhalation, and noses and lungs were removed. Nasal and lung tissue homogenates were prepared as described previously (65).

### RNA isolation and RT-qPCR on nasal and lung homogenates

Viral RNA was isolated from 140 µL of tissue homogenates with a QIAamp Viral RNA Kit (Qiagen), and 10 µL of RNA was used to synthesize cDNA with a High-Capacity cDNA Reverse Transcription Kit (Thermo Fisher Scientific). HPIV3 genome presence was assessed by RT-qPCR on a QuantStudio 3 for 40 cycles using 2 µL of cDNA template, 1 µL of 18S (4318839, VIC) endogenous control Taqman primer, 1 µL of HPIV3 (Vi06439670_s1, FAM) Taqman primer, 6 µL H_2_O, and 10 µL of Taqpath master mix in each well.

### Statistics

Graphs were generated, and statistical analysis was performed using GraphPad Prism 10. Results are presented as means ± SEM unless otherwise stated. *P* values of <0.05 were considered statistically significant.

## Data Availability

Raw data for all figures, western blots, and plasmid sequences have been deposited in the Dryad repository (DOI: 10.5061/dryad.xd2547dwk). All other relevant data are within the paper and its supplemental material. Materials are available by MTA with the Trustees of Columbia University, NYC. Reagents are available from the corresponding author under a material agreement with Columbia University.

## SUPPLEMENTAL MATERIAL

Supplemental material jvi.02126-25-s0001.docx Figures S1 to S4.

ASM does not own the copyrights to Supplemental Material that may be linked to, or accessed through, an article. The authors have granted ASM a non-exclusive, world-wide license to publish the Supplemental Material files. Please contact the corresponding author directly for reuse.
