# Supplementary material for: Human parainfluenza virus 3 fusion protein cleavage: a key determinant of infection and spread
Source: J Virol. 2026 Jun 3;100(6):e02126-25. doi: 10.1128/jvi.02126-25 (PMC13288654; doi:10.1128/jvi.02126-25)
Supplement: Supplemental material — Figures S1 to S4. [file jvi.02126-25-s0001.docx]

**Supplementary materials for**

**Human parainfluenza virus 3 fusion protein cleavage: a key determinant of infection and spread**

Kyle Stearns, Tara Marcink, Emily Pawlack, Elizabeth B. Sobolik, Matteo Porotto, Alexander L. Greninger, Stefan Niewiesk, Anne Moscona

**FIG S1 In HPIV3 collected directly from infected human subjects, approximately half of the fusion proteins are cleaved.** HPIV3 directly from HPIV3-infected humans without passage was lysed and immunoprecipitated with anti-HPIV3 HN antibodies, resolved by reducing SDS-PAGE, and immunoblotted with an anti-HPIV3 F antibody.

FIG S2 HPIV3 F E108 and HPIV3 F K108 remain infectious after 24 hours in solution or dried.

HPIV3 F E108 (uncleaved F) and HPIV3 F K108 (cleaved F) were incubated at 4^o^C, 22^o^C or 37^o^C for 24 hours either in solution (wet) or dry. Titration was performed in Vero cells in the presence of 0.1µg/ml TPCK-treated trypsin to permit entry of all viable virions. Values are means ± SEM from three separate biological replicates.

FIG S3 Prefusion uncleaved F0 E108 and cleaved F K108 undergo thermal triggering at similar rates in the absence of HN.

Percent of F in prefusion state following 0- to 60-minute incubation at 55 °C. The percent of prefusion F relative to 0-minute time point was detected with (A) 3x1 or (B) PIA174. Values are means ± SEM from at least three separate biological replicates.

FIG S4 Cell-cell fusion with HPIV3 F E108 paired with an HN that continually engages receptor is inhibited by prefusion F stabilizing VHH-Fc (4C06).

Percent fusion mediated by HPIV3 HN, HPIV3 HN D216R expressed with no F (black), no F targeting cells expressing TMPRSS2 *in trans* (grey), F E108 (red), F E108 targeting cells expressing TMPRSS2 *in trans* (green), F K108 (blue), F K108 targeting cells expressing TMPRSS2 *in trans* (purple). Fusion activity measured with VP64-GFP_1-10_ with dCas9-GFP_11_ complementation assay in the presence or absence of 621nM 4C06 VHH-Fc. ****P* ≤ 0.001, *****P* ≤ 0.0001 by one-way ANOVA and Dunnett’s post hoc test. Data are means ± SEM from at least three separate experiments.
